# Supplementary material for: The Evolution and Characterization of the RNA Interference Pathways in Lophotrochozoa
Source: Genome Biol Evol. 2024 May 7;16(5):evae098. doi: 10.1093/gbe/evae098 (PMC11114477; doi:10.1093/gbe/evae098)
Supplement: evae098_Supplementary_Data [file evae098_supplementary_data.zip › Supplementary_materials.zip!/selected_miRNAs/NoGe_selected_miRNAs/mirdeep_pdf_files/NMRB01000795.1_Notospermus_geniculatus_strain_Ushimado_scaffold795,_whole_genome_shotgun_sequence_20957.pdf]

Provisional ID	:	NMRB01000795.1_Notospermus_geniculatus_strain_Ushimado_scaffold795,_whole_genome_shotgun_sequence_20957
Score total	:	8968.8
Score for star read(s)	:	3.9
Score for read counts	:	8963.9
Score for mfe	:	1.6
Score for randfold	:	0
Score for cons. seed	:	-0.6
Total read count	:	17594
Mature read count	:	17478
Loop read count	:	0
Star read count	:	116

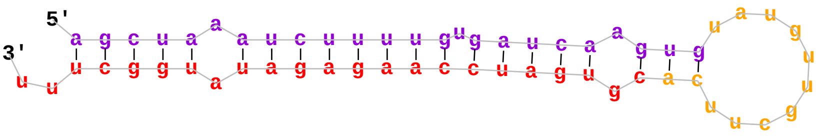

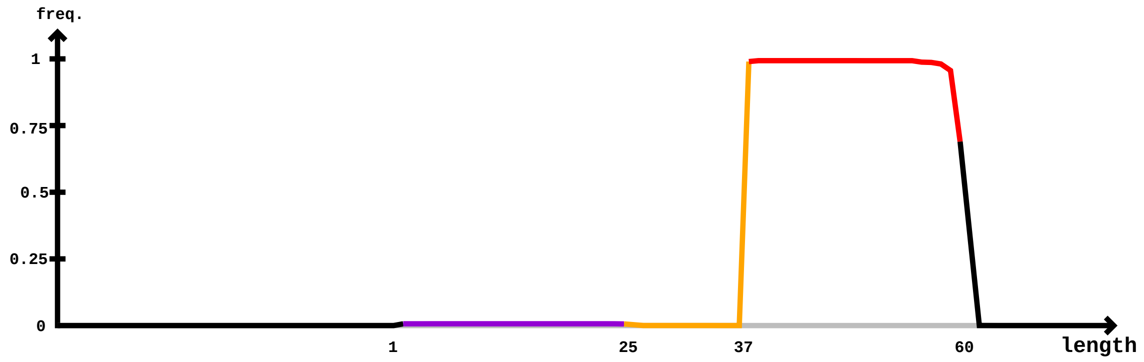

Star Mature

```
5'- cucaaauacacaaguuccaaaagggguguagccacagcuaaaucuuuugugaucaaguguauguugcuucacgugauccaagagauauggcuuuggucaucacccccau  -3'   obs
    cucaaauacacaaguuccaaaagggguguagccacagcuaaaucuuuugugaucaaguguauguugcuucacgugauccaagagauauggcuuuggucaucacccccau         exp
    ......................(((((..((((.(((((.((((((((.(((((.(((..........))).))))))))))))).))))).))))...))))).....   reads    mm    sample
    ...................................agcuaaaucuuuugugaucaag....................................................   1        0     Mm3
    ...................................agcuaaaucuuuugugaucaagu...................................................   4        0     Mm3
    ...................................agcuaaaucuuuugugaucaagug..................................................   28       0     Mm3
    .......................................................................cgugauccaagagauauU....................   1        1     Mm3
    .......................................................................cgugauccaagagauaug....................   17       0     Mm3
    .......................................................................cgugauccaagagauaugU...................   1        1     Mm3
    .......................................................................cgugauccaagagauauggc..................   14       0     Mm3
    .......................................................................cgugauccaagagauauAgcu.................   1        1     Mm3
    .......................................................................Agugauccaagagauauggcu.................   1        1     Mm3
    .......................................................................cgugauccaagagauaGggcu.................   1        1     Mm3
    .......................................................................cgugauccaagagauauggcG.................   1        1     Mm3
    .......................................................................cgugauccaagagauauggcA.................   5        1     Mm3
    .......................................................................cgugauccaaCagauauggcu.................   1        1     Mm3
    .......................................................................cgugauccaagagauauggcC.................   1        1     Mm3
    .......................................................................cgugauccaagagauauggcu.................   84       0     Mm3
    .......................................................................cgugauccaagagauauUgcu.................   1        1     Mm3
    .......................................................................Ngugauccaagagauauggcu.................   1        1     Mm3
    .......................................................................cgugauccaagagauauCgcuu................   7        1     Mm3
    .......................................................................cgugauccaagagauauggcuG................   1        1     Mm3
    .......................................................................cgugauccaagagauauggcuu................   965      0     Mm3
    .......................................................................cgugauccaagagauauAgcuu................   3        1     Mm3
    .......................................................................cgugauccaagagauaGggcuu................   3        1     Mm3
    .......................................................................cgugauccaagagauaAggcuu................   1        1     Mm3
    .......................................................................cgugauccaagagauauUgcuu................   3        1     Mm3
    .......................................................................cgugauccaagagauCuggcuu................   1        1     Mm3
    .......................................................................cUugauccaagagauauggcuu................   1        1     Mm3
    .......................................................................cgugauccGagagauauggcuu................   8        1     Mm3
    .......................................................................cgugauccaagagauauggcCu................   1        1     Mm3
    .......................................................................cgugauccaagagauaugUcuu................   2        1     Mm3
    .......................................................................cgugauccaaCagauauggcuu................   1        1     Mm3
    .......................................................................cgAgauccaagagauauggcuu................   1        1     Mm3
    .......................................................................cgugauccaaAagauauggcuu................   2        1     Mm3
    .......................................................................cgugauccaagagauauggcuuC...............   1        1     Mm3
```

```
                                  Star                                    Mature
cucaaauacacaaguuccaaaaggguguagccacagcuaaaucuuuugugaucaaguguauguugcuucacgugauccaagagauauggcuuuggucaucacccccau
......................................................................cgugauccaaCagauauggcuuu...............  3     1   Mm3
......................................................................cgugaCccaagagauauggcuuu...............  1     1   Mm3
......................................................................cguUauccaagagauauggcuuu...............  1     1   Mm3
......................................................................cgugauccaagagauauggcuuG...............  2     1   Mm3
......................................................................cgugauccaagUgauauggcuuu...............  2     1   Mm3
......................................................................cgugauccaagagauaCggcuuu...............  4     1   Mm3
......................................................................cgugauccaagagauauggcuuA...............  48    1   Mm3
......................................................................cgugauccaagagaCauggcuuu...............  1     1   Mm3
......................................................................cgugauccaagagauaGggcuuu...............  4     1   Mm3
......................................................................Ugugauccaagagauauggcuuu...............  1     1   Mm3
......................................................................cgugauccaagaUauauggcuuu...............  1     1   Mm3
......................................................................cguCauccaagagauauggcuuu...............  1     1   Mm3
......................................................................cgugauccaagagauauggcuuu...............  1743  0   Mm3
......................................................................cgugauccaaAagauauggcuuu...............  2     1   Mm3
......................................................................cgugauccaagagauauAgcuuu...............  16    1   Mm3
......................................................................cgugauccGagagauauggcuuu...............  16    1   Mm3
......................................................................cgugauccaagagauaAggcuuu...............  3     1   Mm3
......................................................................cgugauccaagagauauCgcuuu...............  9     1   Mm3
......................................................................cgugauccaagagauauUgcuuu...............  9     1   Mm3
......................................................................cAugauccaagagauauggcuuu...............  1     1   Mm3
......................................................................Ngugauccaagagauauggcuuu...............  1     1   Mm3
......................................................................cgugauccaagagauauggcuuuU..............  228   1   Mm3
......................................................................cgugauccaagagauauggcuuug..............  1     0   Mm3
......................................................................cgugauccaagagauauggcuuuA..............  23    1   Mm3
......................................................................cgugauccaagagauauggcuuuC..............  1     1   Mm3
.......................................................................gugauccaagagauauggcu.................  1     0   Mm3
.......................................................................gugauccGagagauauggcuuu...............  1     1   Mm3
.......................................................................gugauccaagagauauggcuuu...............  7     0   Mm3
.......................................................................gugauccaagagauauggcuuuU..............  1     1   Mm3

......................................................................cgugauccaagagauauggcu.................  8     0   Fm3
......................................................................cgugauccaagagauauggcuu................  7     0   Fm3
......................................................................cgugauccaagagauauggcuuA...............  1     1   Fm3
......................................................................cgugauccaagagauauggcuuu...............  8     0   Fm3
......................................................................Ggugauccaagagauauggcuuu...............  1     1   Fm3

......................................................................cgugauccaagagauaug....................  39    0   Mm1
......................................................................cgugauccaagagauaugU...................  9     1   Mm1
......................................................................cgugauccaagagauauggc..................  22    0   Mm1
......................................................................Ugugauccaagagauauggc..................  1     1   Mm1
......................................................................cgugauccaagagauauggcu.................  80    0   Mm1
......................................................................cgugauccaagagauauAgcu.................  1     1   Mm1
......................................................................cgugauccaagagauauCgcuu................  7     1   Mm1
......................................................................cgugauccaagagauGuggcuu................  2     1   Mm1
......................................................................Ugugauccaagagauauggcuu................  1     1   Mm1
......................................................................cguAauccaagagauauggcuu................  1     1   Mm1
......................................................................Ngugauccaagagauauggcuu................  1     1   Mm1
......................................................................cgugaucUaagagauauggcuu................  1     1   Mm1
......................................................................cgugauccaagagauauggcuu................  836   0   Mm1
......................................................................cgugauccaagagauaCggcuu................  2     1   Mm1
......................................................................cgugauccaagagGuauggcuu................  1     1   Mm1
......................................................................cgugauccaagagauaGggcuu................  1     1   Mm1
......................................................................cgugauccaagagauauAgcuu................  2     1   Mm1
......................................................................cgugauccaagagauaAggcuu................  5     1   Mm1
......................................................................cgugauccCagagauauggcuu................  1     1   Mm1
......................................................................cgugauccaagagauauUgcuu................  6     1   Mm1
......................................................................cgugauccaaCagauauggcuu................  1     1   Mm1
......................................................................cgugauccaagagauauUgcuuu...............  7     1   Mm1
......................................................................cgugauccaagagauaGggcuuu...............  1     1   Mm1
......................................................................cgCgauccaagagauauggcuuu...............  1     1   Mm1
......................................................................cgugauccaagagauGuggcuuu...............  4     1   Mm1
......................................................................cgugauccaagagauauAgcuuu...............  4     1   Mm1
......................................................................cgugauccaagagauauggcuuu...............  928   0   Mm1
......................................................................cgugauccaaAagauauggcuuu...............  3     1   Mm1
......................................................................cgugauccaagagauauggcuuA...............  52    1   Mm1
......................................................................cgugauccaagagauauggcuuG...............  1     1   Mm1
......................................................................cgugaucUaagagauauggcuuu...............  2     1   Mm1
......................................................................cgugauccaagUgauauggcuuu...............  1     1   Mm1
......................................................................cgugauccaagagauauCgcuuu...............  2     1   Mm1
......................................................................cgugauccGagagauauggcuuu...............  18    1   Mm1
```

Star   Mature

```
cucaaauacacaaguuccaaaaggguguagccacagcuaaaucuuuugugaucaaguguauguugcuucacgugauccaagagauauggcuuuggucaucacccccau
......................................................................cgugauccaagagauaCggcuuu...............   2     1   Mm1
......................................................................cgugaNccaagagauauggcuuu...............   1     1   Mm1
......................................................................cgugauccaagagauaAggcuuu...............   1     1   Mm1
......................................................................cgugauUcaagagauauggcuuu...............   3     1   Mm1
......................................................................cgugauccaagagauauggcuuuU..............   59    1   Mm1
......................................................................cgugauccaagagauauggcuuuA..............   28    1   Mm1
.......................................................................gugauccaagagauauggcuuu...............   6     0   Mm1

..................................agcuaaaucuuuugugaCcaagu...................................................   1     1   Mm2
..................................agcuaaaucuuuugugaucaagu...................................................   4     0   Mm2
..................................agcuaaaucuuuugugUucaagug..................................................   1     1   Mm2
..................................agcuaaaucuuuugugaucaagug..................................................   65    0   Mm2
..................................agcuaaaucuuuugugaucaagugu.................................................   2     0   Mm2
..................................agcuaaaucuuuugugaucaaguguauguugcuuca......................................   1     0   Mm2
.....................................uaaaucuuuugugaucaaguguauguugcu.........................................   1     0   Mm2
..........................................................uauguugcuucacgugauccaagU..........................   1     1   Mm2
......................................................................cgugauccaagagauaug....................   38    0   Mm2
......................................................................cgugauccaaUagauaug....................   1     1   Mm2
......................................................................cgugauccaagagauaugU...................   3     1   Mm2
......................................................................cgugauccaagagauaugg...................   6     0   Mm2
......................................................................cgugauccaagagauauUgc..................   2     1   Mm2
......................................................................cgugauccaagagauauggc..................   61    0   Mm2
......................................................................cguUauccaagagauauggc..................   1     1   Mm2
......................................................................cgugauccaagagauauggcA.................   3     1   Mm2
......................................................................cgugauccaagagauauggcu.................   227   0   Mm2
......................................................................cgugauccaagagauauUgcu.................   2     1   Mm2
......................................................................cgugauccaagagauaCggcu.................   1     1   Mm2
......................................................................cgugauccaagagauauAgcu.................   1     1   Mm2
......................................................................cgugauccaagagauauCgcu.................   2     1   Mm2
......................................................................cgugauccaagagauauggcG.................   2     1   Mm2
......................................................................Ugugauccaagagauauggcu.................   1     1   Mm2
......................................................................cgugauccaagagauaGggcu.................   1     1   Mm2
......................................................................cgugauccaaUagauauggcu.................   1     1   Mm2
......................................................................cgugauccaaCagauauggcu.................   1     1   Mm2
......................................................................cgugauccGagagauauggcu.................   1     1   Mm2
......................................................................cguAauccaagagauauggcu.................   1     1   Mm2
......................................................................cgugauccaagagGuauggcuu................   1     1   Mm2
......................................................................cgugauccaaUagauauggcuu................   4     1   Mm2
......................................................................Ngugauccaagagauauggcuu................   3     1   Mm2
......................................................................cgugaucAaagagauauggcuu................   1     1   Mm2
......................................................................cgugauccaagagauauggcAu................   2     1   Mm2
......................................................................cgugauccaagagauauggcuu................   2695  0   Mm2
......................................................................cgugauccaagagauauUgcuu................   7     1   Mm2
......................................................................cgugauccaagagaCauggcuu................   1     1   Mm2
......................................................................cgugauccaagagauauggcuG................   8     1   Mm2
......................................................................cgugauccaagagauauggcuC................   1     1   Mm2
......................................................................cgugaCccaagagauauggcuu................   1     1   Mm2
......................................................................cgugauccaaCagauauggcuu................   2     1   Mm2
......................................................................cgugauccaagagauauAgcuu................   19    1   Mm2
......................................................................cguCauccaagagauauggcuu................   1     1   Mm2
......................................................................cgugauccaagagCuauggcuu................   2     1   Mm2
......................................................................cgugGuccaagagauauggcuu................   1     1   Mm2
......................................................................cgugauccaagagauaAggcuu................   6     1   Mm2
......................................................................cgugauAcaagagauauggcuu................   1     1   Mm2
......................................................................cgugaucGaagagauauggcuu................   1     1   Mm2
......................................................................cgAgauccaagagauauggcuu................   1     1   Mm2
......................................................................cgugauccCagagauauggcuu................   1     1   Mm2
......................................................................cgugauccaagagauGuggcuu................   2     1   Mm2
......................................................................cgugauccaagagauauggcuA................   2     1   Mm2
......................................................................cgugauccaagagauaGggcuu................   2     1   Mm2
......................................................................cgugauccGagagauauggcuu................   5     1   Mm2
......................................................................cgugauccaagagauauggUuu................   2     1   Mm2
......................................................................cgugauccaagagauauCgcuu................   16    1   Mm2
......................................................................cgugauccaagagauaCggcuu................   13    1   Mm2
......................................................................cgugauccaaAagauauggcuu................   2     1   Mm2
......................................................................cUugauccaagagauauggcuu................   1     1   Mm2
......................................................................cgugauccaagagauUuggcuuu...............   1     1   Mm2
......................................................................cgugauccaagagauGuggcuuu...............   4     1   Mm2
......................................................................cgugauccaagagauaGggcuuu...............   15    1   Mm2
......................................................................cgugauccaagCgauauggcuuu...............   1     1   Mm2
```

Star Mature

```
cucaaauacacaaguuccaaaaggguguagccacagcuaaaucuuuugugaucaaguguauguugcuucacgugauccaagagauauggcuuuggucaucacccccau
......................................................................cgugauccaagagauaAggcuuu...............  17     1   Mm2
......................................................................cgugauccaagagauauggcuuC...............  3      1   Mm2
......................................................................cgugauccaagaAauauggcuuu...............  2      1   Mm2
......................................................................cgugauccaagagauauAgcuuu...............  52     1   Mm2
......................................................................cgugauAcaagagauauggcuuu...............  1      1   Mm2
......................................................................cgugaucGaagagauauggcuuu...............  1      1   Mm2
......................................................................cgugauccaaUagauauggcuuu...............  14     1   Mm2
......................................................................cgugauccaagagauauCgcuuu...............  42     1   Mm2
......................................................................cgugauccaUgagauauggcuuu...............  3      1   Mm2
......................................................................cgugauccaagGgauauggcuuu...............  2      1   Mm2
......................................................................Ugugauccaagagauauggcuuu...............  8      1   Mm2
......................................................................cUugauccaagagauauggcuuu...............  1      1   Mm2
......................................................................cgugauccaagagUuauggcuuu...............  2      1   Mm2
......................................................................cgugauccaagagauauggcuCu...............  2      1   Mm2
......................................................................cgugauccaagagCuauggcuuu...............  3      1   Mm2
......................................................................cgugauccaagaUauauggcuuu...............  1      1   Mm2
......................................................................cgugauccGagagauauggcuuu...............  39     1   Mm2
......................................................................cgugaucUaagagauauggcuuu...............  5      1   Mm2
......................................................................cgugaCccaagagauauggcuuu...............  1      1   Mm2
......................................................................cgugauccaagagauauggcuuG...............  5      1   Mm2
......................................................................Ggugauccaagagauauggcuuu...............  1      1   Mm2
......................................................................cgugauccaagagauauUgcuuu...............  30     1   Mm2
......................................................................cgugGuccaagagauauggcuuu...............  1      1   Mm2
......................................................................cgugauUcaagagauauggcuuu...............  2      1   Mm2
......................................................................Ngugauccaagagauauggcuuu...............  5      1   Mm2
......................................................................cgugauccaaCagauauggcuuu...............  3      1   Mm2
......................................................................cgugauccaagagauaCggcuuu...............  22     1   Mm2
......................................................................cCugauccaagagauauggcuuu...............  1      1   Mm2
......................................................................cgugauccUagagauauggcuuu...............  1      1   Mm2
......................................................................cgugauccaagagauauggcuuA...............  90     1   Mm2
......................................................................cgugUuccaagagauauggcuuu...............  2      1   Mm2
......................................................................cguAauccaagagauauggcuuu...............  2      1   Mm2
......................................................................cgugauccaagagaGauggcuuu...............  3      1   Mm2
......................................................................cAugauccaagagauauggcuuu...............  1      1   Mm2
......................................................................cgugauccaagagaAauggcuuu...............  1      1   Mm2
......................................................................cgugauccaGgagauauggcuuu...............  2      1   Mm2
......................................................................cgugaNccaagagauauggcuuu...............  1      1   Mm2
......................................................................cgCgauccaagagauauggcuuu...............  3      1   Mm2
......................................................................cgugauccaagagauauggcuuu...............  7454   0   Mm2
......................................................................cgugauccCagagauauggcuuu...............  1      1   Mm2
......................................................................cgugaucAaagagauauggcuuu...............  1      1   Mm2
......................................................................cgugauccaaAagauauggcuuu...............  14     1   Mm2
......................................................................cgugauccaagagauauggcuuuC..............  2      1   Mm2
......................................................................cgugauccaagagauauggcuuug..............  1      0   Mm2
......................................................................cgugauccaagagauauCgcuuug..............  1      1   Mm2
......................................................................cgugauccaagagauauggcuuuA..............  41     1   Mm2
......................................................................cgugauccaagagauauggcuuuU..............  871    1   Mm2
......................................................................cgugauccaagagauauggcuuugU.............  1      1   Mm2
......................................................................cgugauccaagagauauggcuuuAgu............  2      1   Mm2
.......................................................................gugauccaagagauauggc..................  1      0   Mm2
.......................................................................gugauccaagagauauggcuu................  6      0   Mm2
.......................................................................gugauccaagagauauggcuuu...............  33     0   Mm2
.......................................................................gugauccaagagauaugCcuuu...............  1      1   Mm2
.......................................................................gugauccaagagauauggcuuuU..............  2      1   Mm2

......................................................................cgugauccaagagauauggcuuG...............  1      1   Fm1
......................................................................cgugauccaagagauauggcuuu...............  31     0   Fm1
......................................................................Agugauccaagagauauggcuuu...............  1      1   Fm1
......................................................................cgugauccGagagauauggcuuu...............  3      1   Fm1
......................................................................cgugauccaagagauauggcuuuU..............  6      1   Fm1

..................................agcuaaaucuuuugugaucaagug..................................................  10     0   Fm2
......................................................................cgugauccGagagauauggcuu................  9      1   Fm2
......................................................................cgugauccaagagauauggcuuu...............  7      0   Fm2
```
